# Sensitivity to change and association of three-dimensional meniscal measures with radiographic joint space width loss in rapid clinical progression of knee osteoarthritis

**DOI:** 10.1007/s00330-017-5140-1

**Published:** 2017-11-24

**Authors:** Melanie Roth, Katja Emmanuel, Wolfgang Wirth, C. Kent Kwoh, David J. Hunter, Felix Eckstein

**Affiliations:** 1 Institute of Anatomy, Paracelsus Medical University Salzburg & Nuremberg, Strubergasse 21, 5020 Salzburg, Austria; 2 Department of Orthopedics and Traumatology, Paracelsus Medical University, Müllner Hauptstraße 48, 5020 Salzburg, Austria; 3 Chondrometrics GmbH, Ulrichshöglerstrasse 23, 83404 Ainring, Germany; 4 Division of Rheumatology, University of Arizona and University of Arizona Arthritis Center, 1501 N. Campbell Ave, Tucson, AZ USA; 5 Rheumatology Department, Royal North Shore Hospital and Institute of Bone and Joint Research, University of Sydney, Sydney, New South Wales NSW 2006 Australia

**Keywords:** Knee osteoarthritis, Joint space width, Meniscus, Magnetic resonance imaging, Cartilage thickness

## Abstract

**Objective:**

To determine whether 3D meniscal measures had similar sensitivity to longitudinal change as cartilage thickness; to what extent these measures are associated with longitudinal joint space width (JSW) change; and whether the latter associations differ between minimum (mJSW) and fixed-location JSW.

**Methods:**

Two-year changes in medial meniscal position and morphology, cartilage thickness (MRI) and minimum and fixed-location JSW (radiography) were determined in 35 Osteoarthritis Initiative knees [12 men, age: 67 (51-77) years; 23 women, age: 65 (54-78) years], progressing from baseline Kellgren-Lawrence grade ≤2 to knee replacement within 3-5 years. Multiple linear regression assessed the features contributing to JSW change.

**Results:**

Meniscal measures, cartilage thickness and JSW displayed similar sensitivity to change (standardised response mean≤|0.76|). Meniscal changes were strongly associated with JSW change (r≤|0.66|), adding ≤20% to its variance in addition to cartilage thickness change. Fixed-location JSW change (multiple r^2^=72%) was more strongly related to cartilage and meniscal change than mJSW (61%). Meniscal morphology explained more of fixed-location JSW and meniscal position more of mJSW.

**Conclusion:**

Meniscal measures provide independent information in explaining the variance of radiographic JSW change. Fixed-location JSW appears to be more reflective of structural change than mJSW and, hence, a potentially superior measure of structural progression.

***Key Points*:**

• *3D positional/morphological meniscal measures change in rapidly progressing knees.*

• *Similar sensitivity to 2-year change of quantitative meniscal/cartilage measures in rapid progression.*

• *Changes in meniscal measures are strongly associated with radiographic JSW change.*

• *Meniscal change provides information to explain JSW variance independent of cartilage.*

• *Fixed-location JSW reflects structural disease stage more closely than minimum JSW.*

## Introduction

Radiographic joint space width (JSW) is the structural measure currently accepted by regulatory agencies for testing disease-modifying drugs in knee osteoarthritis (KOA) [1]. Radiographic JSW obtained from high-quality X-ray acquisitions has been proven to be sensitive to change in KOA, particularly before knee replacement (KR) [2]. Quantitative reduction in JSW – or an increase in the semi-quantitative joint space narrowing (JSN) grade – is commonly interpreted as a surrogate of cartilage loss. Several, mainly cross-sectional, studies have suggested that JSW measures not only reflect cartilage thickness, but also meniscal properties, in particular meniscal extrusion [3–8]. More importantly, novel measures of radiographic JSW measured at defined (fixed) locations have recently been developed [9] and have been suggested to be more sensitive to change in KOA than the traditional minimum JSW (mJSW) [9–12]. However, it is unknown to what extent the longitudinal change in fixed-location JSW is more or less reflective of change in meniscal morphology, meniscal position, and cartilage morphology than mJSW.

A quantitative analysis technique for obtaining three-dimensional (3D) measures of meniscal position (i.e. extrusion) and morphology from magnetic resonance (MR) images has become available over the past few years [13–15]. Applying this 3D method in cross-sectional analyses, we were able to show that these measures independently contribute to explaining variance in radiographic JSW in healthy reference subjects [16] and also to explaining within-person variance in patients with unilateral JSN [3]. Recently, these 3D measures of meniscal position and morphology have been shown to be sensitive to change in longitudinal progression of KOA in knees with baseline JSN [17]. No study has previously addressed whether longitudinal changes in these novel 3D measures are more or less sensitive to change than cartilage thickness, to what extent they are associated with longitudinal change in JSW, and whether the associations with JSW differ between minimum and fixed-location JSW, however.

The objectives of this study were to determine the sensitivity to change of 3D quantitative meniscal measures in rapidly progressing KOA relative to that of articular cartilage thickness loss, to assess the contribution of longitudinal changes in the 3D meniscal and cartilage thickness measures to those in radiographic JSW, and to examine whether the latter associations differed between minimum and fixed-location radiographic JSW.

## Material and methods

### Study design

The Osteoarthritis Initiative (OAI) is a multicentre, prospective observational cohort study of 4,796 subjects, designed to identify biomarkers and risk factors for KOA incidence and progression [18]. The OAI was approved by the institutional review board of the University of California, San Francisco, as well as each OAI Clinical Center, all patients provided informed consent [18]. OAI participants were 45-79 years of age at enrolment and had (or were at risk of) symptomatic KOA in at least one knee. Clinical data, 3Tesla MR images (Siemens Magnetom Trio, Erlangen, Germany; quadrature transmit-receive knee coils from USA Instruments, Aurora, OH, USA) and radiographs of the knee were acquired at annual visits [18–20].

**Fig. 1:**
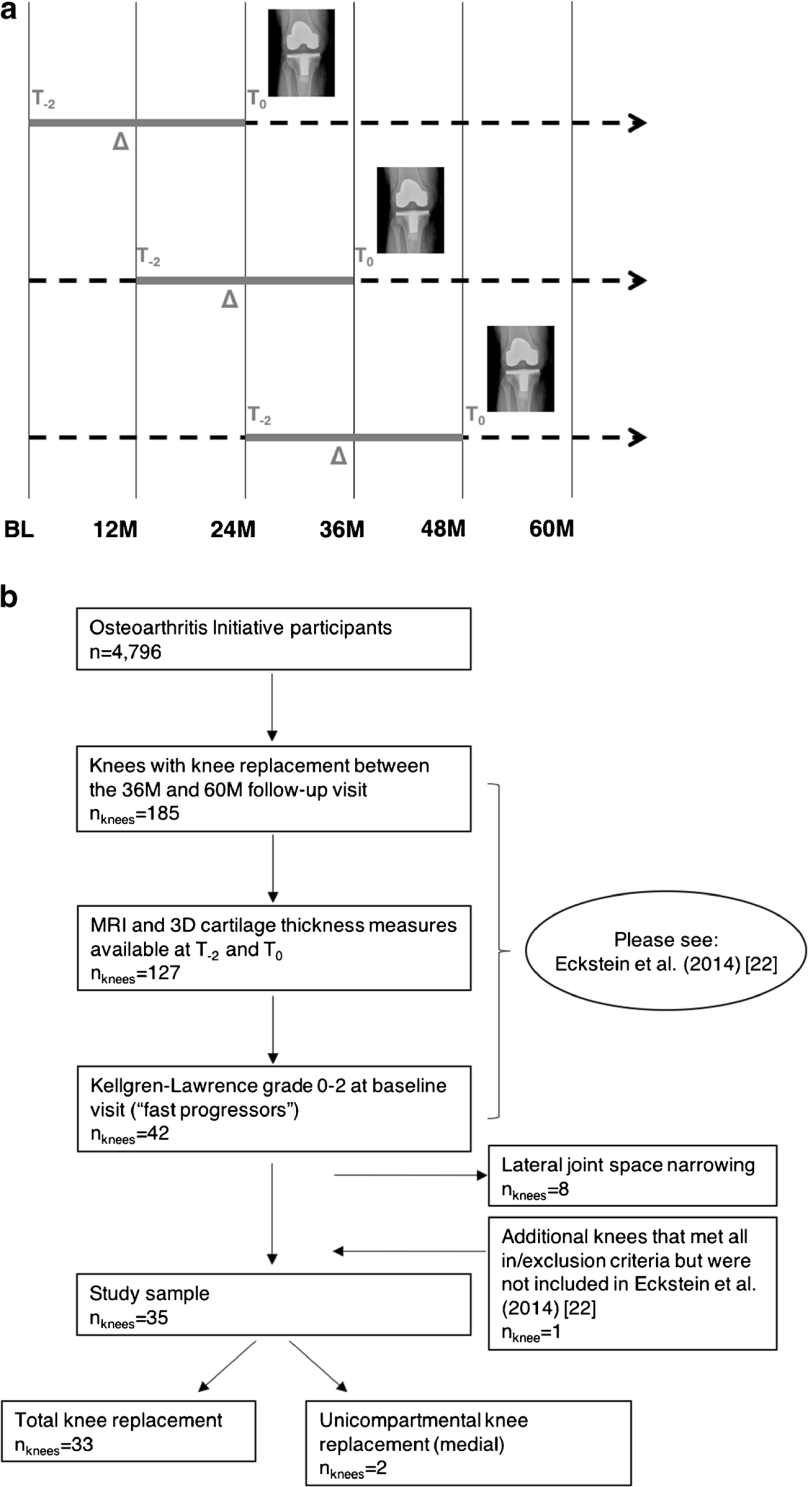
study design and participant selection. **a** study design depicting the two-year period (T_-2_ – T_0_) before surgical knee replacement (KR) depending on the time point of KR. Abbreviations: BL = baseline, M = months; **b** flow chart depicting the participant selection from the Osteoarthritis Initiative database. Abbreviations and definition of T_-2_ and T_0_: see 1a

To be eligible as a case for the current study, total or unicompartmental medial KR had to be confirmed by radiography or by hospital records at the 36-month (M), 48M, or 60M follow-up visit. Further, 3T MR images for which quantitative cartilage analysis [21, 22] had been performed, had to be available for the annual visit before KR (T_0_) and the annual visit two years before T_0_ (T_-2_) (Fig. 1). KRs detected at the 12M or 24M follow-up visit were not included, due to an insufficient longitudinal observation period before KR. If both knees of one participant were reported as replaced at the same, or different time points, both knees were included in the analyses. Only knees with baseline Kellgren-Lawrence grade (KLG) ≤2 were included to ensure rapid and clinically relevant progression of KOA [21, 22]. Knees with lateral Osteoarthritis Society International JSN grades >0 were excluded from the analyses, since lateral compartment JSN increases the risk of lateral progression, potentially masking progression in the medial compartment [12].

### Quantitative joint space width measures

**Fig. 2:**
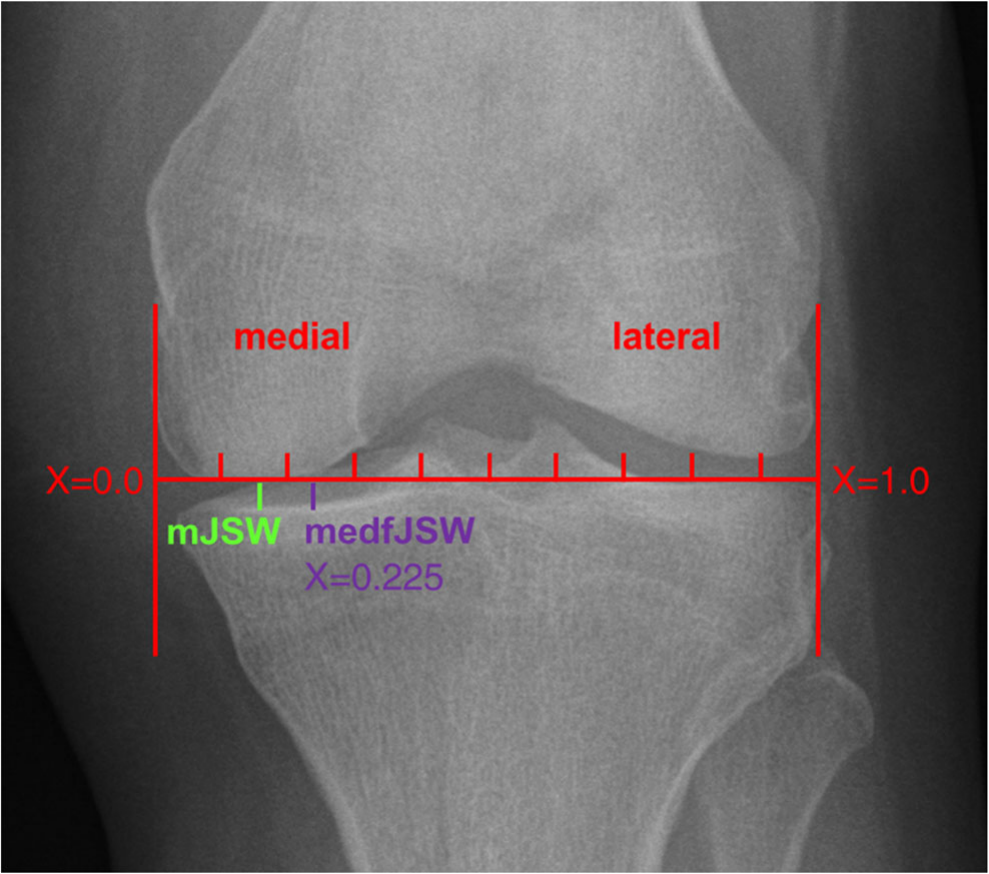
radiographic joint space width. Fixed-flexion radiograph depicting the medial to lateral coordinate system used for fixed-location measurement (medfJSW: medial fixed joint space width at 22.5%), as well as the minimum medial joint space width (mJSW)

Weight-bearing, posterior-anterior fixed-flexion (10°) radiographs were acquired from OAI participants at each annual visit [18, 23]. Semi-automated radiographic JSW measurements were performed for the majority of these knees [9, 10] (Fig. 2). In the current study, we analysed medial compartment mJSW and medial compartment fixed-location JSW at 22.5% of the mediolateral width of the distal femur [medial fixed-location (medf)JSW, Fig. 2] [9] as this measure has been previously shown to display greater sensitivity to change in KOA [10, 24] and a stronger relationship with subsequent KR than mJSW [2].

### Quantitative cartilage thickness measures

**Fig. 3:**
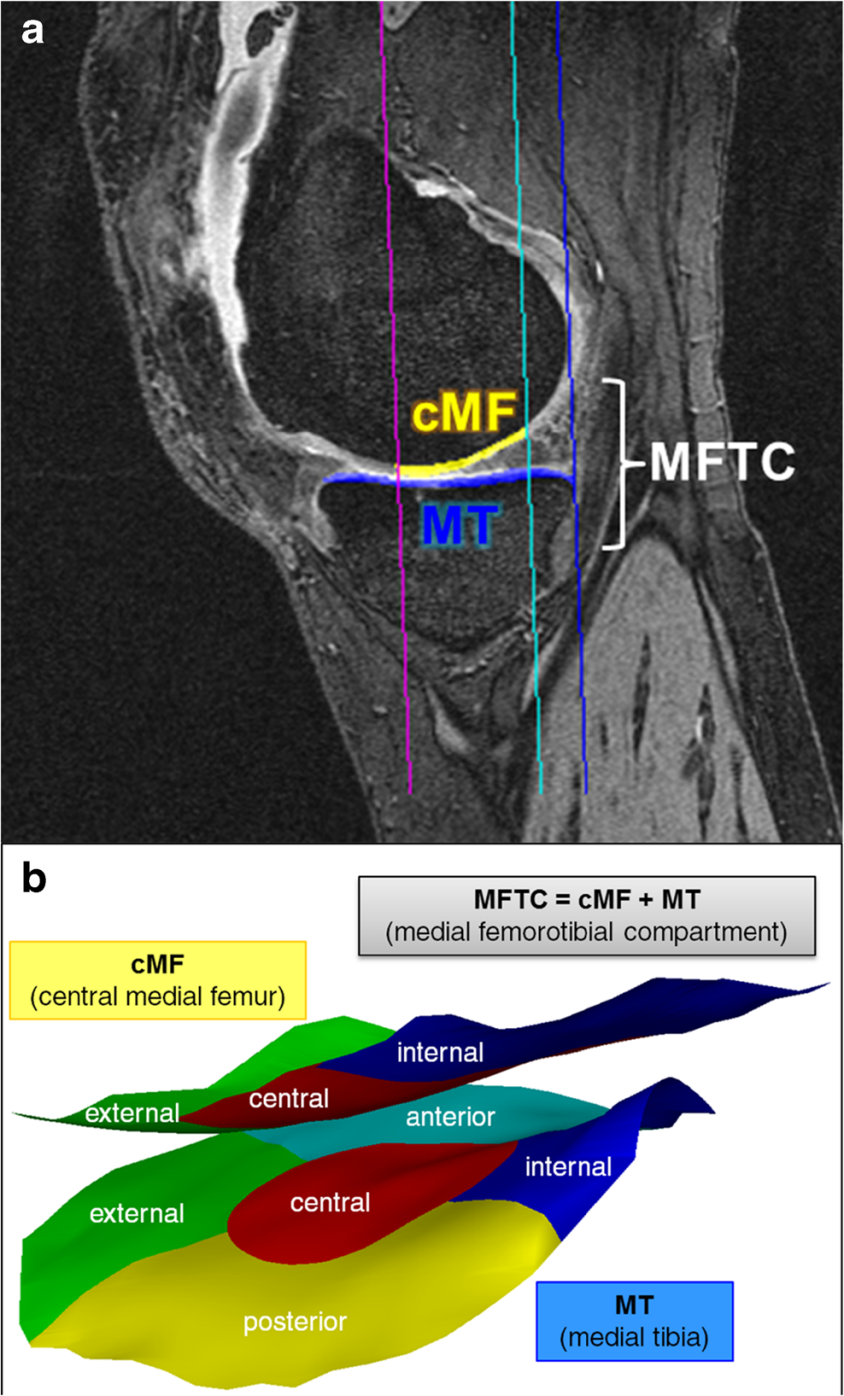
3D analysis of cartilage thickness. MR imaging based, quantitative 3D analysis of the cartilage in the medial femorotibial compartment (MFTC): **a** sagittal MR image from a double echo steady state sequence (with water excitation) with MFTC cartilage segmentation (cMF = central medial femur; MT = medial tibia); **b** 3D reconstruction of the medial tibial and weight-bearing femoral cartilage including subregions

MR images from a sagittal double echo steady state sequence with water excitation (DESSwe, 0.7mm slice thickness; interpolated in-plane resolution 0.37mm × 0.37mm) [19] were used for segmenting the femorotibial cartilages at the T_-2_ and T_0_ visits [22] (Fig. 3a). Total subchondral bone area and the cartilage joint surface area of the medial tibia (MT) and the central (weight-bearing) medial femoral condyle (cMF) were manually traced by experienced readers, as described previously [22]. The analysis, conducted with blinding to acquisition order, relied on custom software (Chondrometrics GmbH, Ainring, Germany). Quality control readings were performed by an expert reader. The mean cartilage thickness was computed for MT and cMF, for the total medial femorotibial compartment (MFTC=MT+cMF), and for a combined central femorotibial subregion (cMFTC) (Fig. 3b) [25, 26].

### Quantitative meniscal measures

**Fig. 4:**
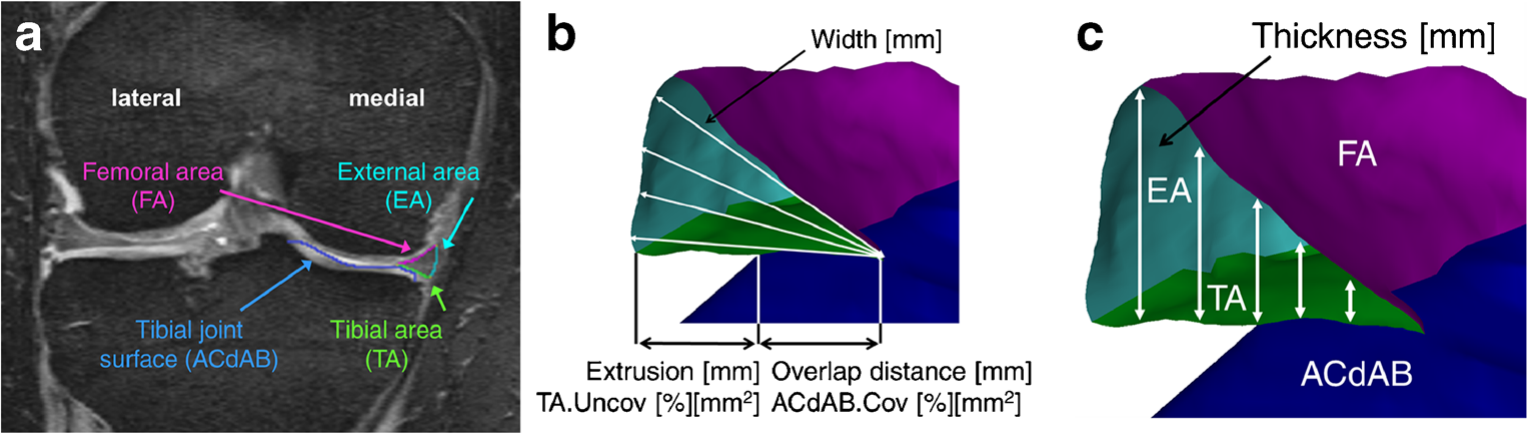
3D analysis of the menisci. MR imaging based, quantitative 3D analysis of the menisci in the medial compartment: **a** coronal MR image from a double echo steady state sequence (with water excitation) with segmentation of the medial meniscus; **b**–**c** 3D reconstruction of the medial meniscus depicting: ACdAB (joint surface of the tibia, consisting of the area of the cartilage surface, and denuded areas of subchondral bone if applicable); the femoral (FA), tibial (TA), and external surface (EA) of the medial meniscus; meniscal thickness and width; overlap area between TA and ACdAB (ACdAB.Cov), uncovered area of the TA (TA.Uncov), overlap distance (OvD) and extrusion distance (Ex)

Coronal multi-planar reconstructions (1.5mm slice thickness; interpolated in-plane resolution 0.37mm × 0.37mm) were derived from the near isotropic sagittal DESSwe MR images. The medial tibial plateau area (i.e. the area of cartilage surface including denuded areas of subchondral bone) and the surfaces of the medial meniscus (tibial, femoral and external area) were segmented at T_-2_ and T_0_ from the coronally reconstructed images obtained, which were previously used for the cartilage thickness measurements (with blinding to acquisition order, Fig. 4a). The segmentations were performed by a PhD (M.R.), who was first formally trained in quantitative meniscus analysis; all segmentations done in this study were quality controlled by an expert reader (K.E.) with >5 years of experience in quantitative meniscus analysis. Each case (two time points, two menisci) required approximately 2.5 h, including quality control readings and potential corrections following quality control. Measures of meniscal morphology included mean and maximal meniscal thickness and width, and meniscal volume (Fig. 4b-c) [13]. Measures of meniscal position relative to the tibial plateau encompassed the tibial plateau area covered by the meniscus (absolute/percent), the tibial meniscal surface area not covering the tibial plateau (absolute/percent), mean and maximal extrusion distance and overlap distance between the meniscus and the medial tibial plateau area (Fig. 4b) [13].

### Statistical analysis

Paired *t*-tests were used to determine whether significant changes in JSW, cartilage and meniscal parameters occurred between T_-2_ and T_0_. The bootstrap method [27] (1000 replications, BCa method, simple sampling), that renders *P* values relatively robust against non-normally distributed data, was applied to use the same statistical approach for all variables, even in the case of non-normal distribution. The Wilcoxon signed-rank test was additionally used to confirm the results for non-normally distributed variables. Sensitivity to change of the parameters was assessed using the standardized response mean (SRM=mean change/standard deviation of change).

Bivariate correlation coefficients (*r*; Pearson; two-sided; bootstrapping as above) between the 2-year change in JSW parameters (mJSW, medfJSW) and the two-year change in meniscal and cartilage measures were assessed.

Multiple linear regression analyses were performed to examine the association of meniscal and cartilage measures with JSW change based on a hierarchical approach, the variables being predefined manually by the study team: Step 1 forced cMFTC cartilage thickness (Fig. 3b) into the model, because this cartilage parameter has previously displayed high sensitivity to change in medial KOA [21]. Please note that in correlation analyses cMFTC cartilage thickness also displayed the strongest coefficient between JSW change amongst the cartilage measures (Table 1). Step 2 allowed the model to include one positional (tibial meniscal surface area not covering the tibial plateau; Fig. 4b) and two morphological (maximal thickness, mean width; Fig. 4b-c) meniscal measures in a stepwise fashion. The specific meniscal measures were chosen based on their high correlation with JSW parameters and their responsiveness (SRM), and so that different meniscal properties were represented in the model. In step 3, the model was allowed to include age (at T_-2_) and body weight change (from T_-2_ to T_0_) in a stepwise manner to compensate for possible confounding. The Akaike information criterion (AIC) permitted comparison of the models at the different stages. For statistical comparison of the model steps, analysis-of-variance was performed. For exploratory purposes the models were also calculated based on a second approach, which exchanged step 1 and 2 in their order. *p* values of < 0.05 were considered significant, no correction was performed for multiple testing due to the exploratory nature of this study.

**Table 1:** Joint space width, cartilage and medial meniscal parameters at 2 years prior to knee replacement (T_-2_) and respective 2-year change (T_-2_→T_0_).

		n	T_-2_ mean±s.d.	Change (T_-2_→T_0_) mean (95%CI)	SRM	r between ∆ mJSW and ∆ of respective parameter	r between ∆ medfJSW and ∆ of respective parameter
Joint space width	mJSW [mm]	28	4.38±1.12	-0.97 (-1.44; -0.49)***	-0.79		
	medfJSW [mm]	29	5.38±1.00	-1.17 (-1.62; -0.72) ***	-1.00		
Cartilage thickness	MFTC [mm]	35	3.51±0.64	-0.38 (-0.56; -0.19) **	-0.70	0.65**	0.68**
	cMFTC [mm]	35	4.40±0.84	-0.72 (-1.03; -0.40) **	-0.77	0.70**	0.73**
	MT [mm]	35	1.74±0.27	-0.13 (-0.18; -0.07) **	-0.83	0.63**	0.64**
	cMF[mm]	35	1.77±0.43	-0.25 (-0.39; -0.11) **	-0.62	0.64**	0.67**
Medial meniscus	ACdAB.Cov% [%]	35	45.47±7.97	-6.06 (-8.80; -3.32) **	-0.76	0.58**	0.53**
	ACdAB.Cov [mm^2^]	35	463.8±101.5	-54.7 (-82.8; -26.6) **	-0.67	0.39*	0.38*
	TA.Uncov% [%]	35	19.5±9.90	6.7 (3.2; 10.1) **	0.67	-0.60**	-0.46*
	TA.Uncov [mm^2^]	35	103.28±54.5	29.83 (13.30; 46.35) **	0.62	-0.66**	-0.49**
	Ex.mean [mm]	35	2.60±1.54	0.80 (0.29; 1.32) **	0.54	-0.56**	-0.50**
	Ex.max [mm]	35	6.64±1.33	0.59 (0.30; 0.87) **	0.70	-0.40*	-0.35
	OvD.mean [mm]	35	10.33±1.82	-1.35 (-0.73; -1.96) **	0.75	-0.55**	-0.51**
	OvD.min [mm]	35	3.35±1.85	-0.71 (-0.19; -1.22) *	0.47	-0.59**	-0.34
	Wid.mean [mm]	35	8.73±1.07	-0.49 (-0.74; -0.24) **	-0.67	0.47*	0.53**
	Wid.max [mm]	35	15.10±2.09	-1.14 (-1.87; -0.41) **	-0.53	0.19	0.37*
	Th.mean [mm]	35	2.60±0.37	0.06 (0.00; 0.13)	0.33	-0.21	-0.48**
	Th.max [mm]	35	6.63±1.11	0.27 (0.02; 0.53) *	0.37	-0.43*	-0.62**
	Volume [mm^3^]	35	2018.9±546.5	6.1 (-70.8; 83.1)	0.03	-0.17	-0.38*

*Abbreviations*: *CI* confidence interval; change from paired sample t-test bootstrap BCa; *SRM* standardized response mean; r: correlation coefficient (Pearson, bootstrap BCa method, 1000 replications); *p*-value: *… *p* < 0.05; **… *p* < 0.01; ***… *p* < 0.001; mJSW = minimum joint space width; medfJSW = medial fixed-location joint space width; (c)MFTC = (central) medial femorotibial compartment; MT = medial tibia; cMF = central medial femur; ACdAB.Cov% = percentage of tibial plateau covered by the meniscus; ACdAB.Cov = tibial plateau area covered by the meniscus; TA.Uncov% = percentage of tibial meniscal surface not covering the tibial plateau; TA.Uncov = tibial meniscal surface area not covering the tibial plateau; Ex.mean/max = mean/maximal meniscal extrusion; OvD.mean/max = mean/maximal overlap distance; Wid.mean/max = mean/maximal meniscal width; Th.mean/max = mean/maximal meniscal thickness.

All statistical analyses, except for the AIC (Stata V14.2, StataCorp, TX, USA), were done using SPSS 23 (IBM Corporation, Armonk, NY, USA).

## Results

### Sample description

Thirty-five knees of 33 OAI participants (23 women; baseline age 64.7±7.1, body mass index 30.0±4.0 kg/m^2^, KLG0/1/2: 5/8/22, 33 total/two medial unicompartmental KRs) received a KR between the 36M and 60M follow-up visit (13 at 36M, seven at 48M, and 15 at 60M; Fig. 1). Descriptive statistics are presented in Table 1.

### Changes in the medial meniscus

All meniscal measures, except for meniscal volume and mean thickness, showed significant changes over the two-year observation period before KR. The position of the medial meniscus relative to the tibial plateau changed significantly, with the positional parameters showing a loss in coverage of the tibial plateau by the meniscus. Meniscal width displayed a reduction over time, whereas maximal meniscal thickness increased (Table 1). For non-normally distributed parameters the Wilcoxon signed-rank test displayed the same results (data not shown).

The percent area of the tibial plateau covered by the meniscus and the mean overlap distance were observed to be the most sensitive meniscal measures (*SRM* =  − 0.76/0.75). The majority of the positional measures reached an *SRM* ≥ |0.62|, which was the lowest absolute SRM observed for cartilage measures. Somewhat lower absolute SRMs were observed for morphological meniscal measures. As a reference, the SRM of mJSW change was −0.79 and −1.00 for medfJSW change. The most responsive cartilage measure was MT cartilage thickness change (*SRM* = 0.83 ) (Table 1).

### Correlation analyses

The correlation coefficients of mJSW change with change in meniscal position were generally greater than those with change in meniscal morphology (Table 1). Among meniscal measures, the highest correlation with mJSW change was observed for change in tibial meniscal surface area not covering the tibial plateau (r=-0.66; *p*<0.01); this association was similar in magnitude to that between mJSW change and change in cartilage thickness (0.63≤r≤0.70; *p*<0.01) (Table 1).

For medfJSW, the correlations with change in meniscal measures were observed to be similar for positional and morphological parameters (Table 1). In contrast to mJSW, the strongest correlation among the meniscal parameters with medfJSW change was noted for change in maximal meniscal thickness (r=-0.62; *p*<0.01). The correlations of medfJSW change with change in cartilage thickness measures were observed to be somewhat greater (0.64≤r≤0.73; *p*<0.01) than those of medfJSW change with change in meniscal measures (Table 1).

### Meniscal contribution to change in JSW

In the multiple regression models, 47% of the variance in 2-year mJSW change before KR was explained by change in cMFTC cartilage thickness. Change in meniscal position relative to the tibial plateau added 9% to the explained variance, and baseline age another 5%. This resulted in a total of 61% of explained variance in mJSW change (Table 2).

**Table 2:** Linear model of predictors of two-year change (∆) prior to knee replacement in minimum medial joint space width (∆mJSW) and medial fixed-location joint space width (∆medfJSW).

	b (95% CI)	SE b	*β*	*p*	*r* ^*2*^ (#)	cumulative adjusted *r* ^*2*^ (§)	p ANOVA (&)	AIC
∆mJSW (n=28, ♂= 9, ♀=19)								
Step 1								
Constant	-0.27 (-0.72, 0.17)	0.22		0.219				
cMFTC	0.95 (0.56, 1.34)	0.19	0.70	0.000	0.49	0.47	0.000	74.53
Step 2								
Constant	-0.14 (-0.57, 0.28)	0.21		0.494				
cMFTC	0.65 (0.22, 1.09)	0.21	0.49	0.005	0.49			
TA.Uncov [mm^2^]	-0.01 (-0.02, 0.00)	0.00	-0.38	0.023	0.43	0.56	0.023	70.59
Step 3								
Constant	-3.05 (-5.91, -0.19)	1.39		0.038				
cMFTC	0.60 (0.19, 1.01)	0.20	0.44	0.006	0.49			
TA.Uncov [mm^2^]	-0.01 (-0.02, 0.00)	0.00	-0.35	0.025	0.43			
Age	0.04 (0.00, 0.09)	0.02	0.26	0.045	0.18	0.61	0.045	67.80
∆medfJSW (n=29, ♂= 10, ♀=19)								
Step 1								
Constant	-0.46 (-0.86, -0.05)	0.12		0.030				
cMFTC	0.90 (0.56, 1.34)	0.16	0.73	0.000	0.53	0.52	0.000	72.38
Step 2								
Constant	-0.31 (-0.64, 0.02)	0.16		0.067				
cMFTC	0.65 (0.38, 0.92)	0.13	0.53	0.000	0.53			
Th.max	-0.55 (-0.88, -0.22)	0.16	-0.37	0.002	0.39	0.67	0.001	62.03
Wid.mean	0.43 (0.06, 0.79)	0.18	0.26	0.024	0.28	0.72	0.024	57.97
Step 3								
	

*Abbreviations*: # … respective r^2^ from bivariate correlation; § … cumulative adjusted r^2^ from multiple linear stepwise regression (explained variance); *b* beta; *CI* confidence interval; *SE* standard error; *β* standardized beta; *p* … *p*-value; & … *p*-value from ANOVA from change in r^2^; AIC … Akaike information criterion; others see Table 1.

Change in cMFTC cartilage thickness explained 52% of the variance in medfJSW change. An additional 20% of the variance of medfJSW change was explained by meniscal morphology changes (thickness, width), amounting to a total of 72% variance explained. Unlike for mJSW, the regression model did not include a positional meniscal or demographic measure (Table 2).

Explorative regression analyses, which first included meniscal measures and then cartilage thickness, yielded the same explanatory parameters and the same total variance explained for mJSW (61% = 41% meniscal position change + 15% cMFTC cartilage thickness change + 5% age), as well as for medfJSW (72% = 48% meniscal morphology changes + 24% cMFTC cartilage thickness change).

## Discussion

The results of this study showed that sensitivity to change is similar among positional and morphological meniscal measures, MRI cartilage thickness and radiographic mJSW during a 2-year observation interval before KR. Change in meniscal measures provided significant independent information in explaining the variation in longitudinal changes of both radiographic JSW measures (minimum and fixed-location) when combined with cartilage thickness measures in multiple regression models. Of note, the model for fixed-location JSW change was able to explain more variance than that for mJSW change. Within these models, meniscal extrusion appeared to play a greater role in explaining longitudinal variability of mJSW, and meniscal morphology in explaining that of fixed-location JSW change. Exploratory analyses confirmed this difference in meniscal involvement regarding the two different measures of JSW, and showed that change in meniscal parameters alone can account for up to 48% of the change in JSW parameters in rapidly progressive KOA.

A limitation of the study was the small sample size, although the very strict inclusion criteria (baseline KLG 0-2, KR reported at 36–60-month follow-up) ensured inclusion of a group of “rapid clinical progressors” in whom previous work confirmed very high rates of change in mJSW and fixed-location JSW [2]. As the study included 35 knees of 33 participants, sensitivity analyses limited to one knee per participant were performed, which showed very similar results. Also, the sensitivity to change of meniscal measures was similar to that of cartilage thickness measures in the same sample, over the same observation period. These measures were not only correlated with a traditional measure of radiographic progression (mJSW) but also with one recently shown to display greater sensitivity to change (22.5% fixed-location JSW) than mJSW [10, 24].

Hunter et al. previously showed that meniscal position change accounts for a substantial proportion of mJSW change [4]. Our results lead us to a similar conclusion. Previous work was limited to 2D measures of the meniscus in one coronal and one sagittal slice, assessing meniscal extrusion and height, and the coverage of the tibial plateau each to the nearest millimetre [4]. Although no direct comparisons between 2D and 3D measurements were made in the current study, the 3D approach used provides more comprehensive information on the meniscus. Also, it is potentially more robust, as it is less dependent on specific positioning and orientation of the joint in the scanner, and on the selection of specific slices for analysis [13]. Additionally, the current study used quantitative cartilage measures rather than semi-quantitative cartilage scores and included an analysis of fixed-location JSW which have not been previously examined.

Over the 2 years before KR, the coverage of the medial tibial plateau declined by 6.06% [3.32%; 8.80%], that is an average reduction from 45% to 39%. The coverage in healthy reference subjects amounted to 50% [48%; 51%] [15]. Whether cartilage thickness loss causes meniscal change, or vice versa [28], or whether these two structural pathologies occur “hand in hand” mutually perpetuating themselves is open to speculation.

Interestingly, our study revealed that no change in meniscal volume occurred during rapidly progressing KOA, as meniscal width decreased while meniscal thickness increased. This finding is inconsistent with a previous study on longitudinal change of the meniscus in KOA [17]. A potential explanation is that the longitudinal increase in extrusion is so strong that tissue swelling occurs as the meniscus becomes unloaded outside the joint margin. This mechanism has been previously reported for the lateral meniscus [29], and is supported by a study reporting meniscal hypertrophy in late-stage KOA [30].

With regard to the association of longitudinal changes of 3D meniscal measures with fixed-location vs. mJSW, meniscal measures significantly contribute to explaining variance in both radiographic measures. Interestingly, meniscal extrusion measures appear to be more important predictors of mJSW change, likely because with an increase in meniscal extrusion the location of the minimum in radiographic JSW shifts. Under these conditions, mJSW change may not only reflect an actual reduction in JSW, but also the difference in measurement location. This cannot occur with a fixed-location JSW measure, as the measurement is consistently made at a defined location within the joint. Under these circumstances, morphological features of the meniscus, e.g. width and height, appear to be more important contributors in explaining the longitudinal variance in JSW change than meniscal extrusion. Also, the overall variance explained by meniscal and cartilage measures in the longitudinal change of fixed-location JSW was greater than that in mJSW, suggesting that the underlying structural pathology associated with mJSW change is less well defined than that associated with fixed-location JSW change. This suggests that fixed-location JSW is superior to mJSW when trying to estimate change in cartilaginous and meniscal morphology from change in radiographic JSW. This difference may also explain why fixed-location JSW has been shown to predict KR better than mJSW [2].

In conclusion, quantitative 3D measures of the medial meniscus were shown to display considerable changes in rapidly progressing KOA, with a sensitivity to change similar to that of cartilage thickness measures or mJSW. Meniscal measures provided independent information in explaining the longitudinal variance of both mJSW and fixed-location JSW. These results support the concept that longitudinal change in radiographic JSW represents a composite measure of progression of meniscal and cartilage structural pathology, but not of cartilage alone. The total amount of variance in fixed-location JSW explained by quantitative measures of the meniscus and articular cartilage morphology was greater than that in mJSW, suggesting that the underlying structural pathology associated with mJSW change is less well defined than that associated with fixed-location JSW. Change in mJSW was more strongly associated with change in meniscal extrusion than fixed-location JSW, whereas the latter was more closely associated with change in meniscal morphology. Together these findings suggest that fixed-location JSW may be more reflective of structural change in joint tissue morphology, and hence a potentially superior measure of structural progression, compared with mJSW.

## Abbreviations

KR: Knee replacement
KOA: Knee osteoarthritis
JSW: Radiographic joint space width
JSN: Joint space narrowing
mJSW: Minimum medial joint space width
medfJSW: Medial fixed-location joint space width
